# Population Health Solutions for Assessing Cognitive Impairment in Geriatric Patients

**DOI:** 10.1080/13854046.2018.1517503

**Published:** 2018

**Authors:** William Perry, Laura Lacritz, Tresa Roebuck-Spencer, Cheryl Silver, Robert L Denney, John Meyers, Charles E McConnel, Neil Pliskin, Deb Adler, Christopher Alban, Mark Bondi, Michelle Braun, Xavier Cagigas, Morgan Daven, Lisa Drozdick, Norman L Foster, Ula Hwang, Laurie Ivey, Grant Iverson, Joel Kramer, Melinda Lantz, Lisa Latts, Ana Maria Lopez, Michael Malone, Lori Martin-Plank, Katie Maslow, Don Melady, Melissa Messer, Randi Most, Margaret P Norris, David Shafer, Colin M Thomas, Laura Thornhill, Jean Tsai, Nirav Vakharia, Martin Waters, Tamara Golden

**Affiliations:** 1 National Academy of Neuropsychology (NAN).; 2 NAN.; 3 University of California , San Diego.; 4 UT Southwestern Medical Center.; 5 NAN.; 6 Jefferson Neurobehavioral Group.; 7 NAN.; 8 Missouri Memory Center, Citizens Memorial Healthcare.; 9 University of Illinois at Chicago.; 10 Senior Vice President Network Strategy, Optum of United Health Group.; 11 Epic Systems.; 12 Society for Clinical Neuropsychology (SCN).; 13 American Academy of Clinical Neuropsychology (AACN).; 14 Hispanic Neuropsychological Society (HNS).; 15 Health Systems, Alzheimer's Association.; 16 Pearson.; 17 American Academy of Neurology (AAN).; 18 Center for Alzheimer's Care, Imaging and Research, Department of Neurology , University of Utah.; 19 Geriatric EM Section , American College of Emergency Physicians (ACEP).; 20 Department of Emergency Medicine.; 21 Icahn School of Medicine at Mount Sinai , Geriatric Research Education, and Clinical Center, James J. Peters VAMC Geriatric EM Section.; 22 American College of Emergency Physicians (ACEP).; 23 Collaborative Family Healthcare Association (CFHA).; 24 Neuropsychology Outcome Assessment Laboratory and Director, Massachusetts General Hospital for Children Sports Concussion Program, Harvard Medical School.; 25 International Neuropsychological Society (INS).; 26 American Association of Geriatric Psychiatry (AAGP).; 27 IBM Watson Health.; 28 American College of Physicians (ACP).; 29 Health Equity and Inclusion, University of Utah Health Sciences Center.; 30 Cancer Health Equity, Huntsman Cancer Institute.; 31 University of Utah School of Medicine.; 32 American Geriatrics Society.; 33 Aurora Senior Services, Aurora Health Care.; 34 American Association of Nurse Practitioners (AANP).; 35 The Gerontological Society of America (GSA).; 36 Schwartz/Reisman Emergency Medicine Institute, Mount Sinai Hospital, University of Toronto.; 37 Canadian Association of Emergency Physicians.; 38 International Federation of Emergency Medicine.; 39 Research & Development, Psychological Assessment Resources, Inc. (PAR).; 40 American Board of Professional Neuropsychology (ABN).; 41 American Psychological Association (APA).; 42 Pearson.; 43 Department of Veterans Affairs.; 44 Regulatory Affairs, Alzheimer's Association.; 45 University of Colorado , Denver Health.; 46 Cleveland Clinic.; 47 Clinical Innovation and Thought Leadership, Beacon Health Options.; 48 Golden Bioscience Communications.

**Keywords:** Elderly, Geriatrics, Aging, Population health, Dementia, Cognition

## Abstract

In December 2017, the National Academy of Neuropsychology convened an interorganizational Summit on Population Health Solutions for Assessing Cognitive Impairment in Geriatric Patients in Denver, Colorado. The Summit brought together representatives of a broad range of stakeholders invested in the care of older adults to focus on the topic of cognitive health and aging. Summit participants specifically examined questions of who should be screened for cognitive impairment and how they should be screened in medical settings. This is important in the context of an acute illness given that the presence of cognitive impairment can have significant implications for care and for the management of concomitant diseases as well as pose a major risk factor for dementia. Participants arrived at general principles to guide future screening approaches in medical populations and identified knowledge gaps to direct future research. Key learning points of the summit included:

- recognizing the importance of educating patients and healthcare providers about the value of assessing current and baseline cognition;
- emphasizing that any screening tool must be appropriately normalized and validated in the population in which it is used to obtain accurate information, including considerations of language, cultural factors, and education; and
- recognizing the great potential, with appropriate caveats, of electronic health records to augment cognitive screening and tracking of changes in cognitive health over time.

## Introduction

The United States faces a dramatic demographic shift as its population ages, with important implications for health care delivery and costs (Miquel et al., 2017). Age is a significant risk factor for many medical conditions, including cognitive impairment and dementia, and as the number of people over age 65 increases, the number of people living with these conditions will substantially increase (Prince, Comas-Herrera, Knapp, Guerchet, & Karagiannidou, 2016). Cognitive impairment is of particular concern because it is a clinically dominant comorbidity influencing the presentation and management of concomitant conditions. Furthermore, people with cognitive impairment have been shown to use health care services in general and specifically emergency department (ED) services more frequently and at higher cost than those without cognitive deficits (LaMantia, Stump, Messina, Miller, & Callahan, 2016) and they utilize more outpatient visits compared to those without cognitive impairment (Chung et al., 2014; St-Hilaire, Hudon, Preville, & Potvin, 2017). These individuals also experience complications of co-existing medical conditions and have caregivers who experience substantial burden (Bradford, Kunik, Schulz, Williams, & Singh, 2009). Cognitive impairment, which can be indicative of an acute condition, is often a precursor of dementia, a longer term condition involving the development of impaired cognitive function in multiple domains which has an impact on the individual’s daily life. Focusing on the broad condition of cognitive impairment as well as dementia and Alzheimer’s disease is important given that many causes of cognitive impairment are reversible if identified early.

The expected increase in the number of people with sustained cognitive impairment and dementia portends a substantial economic impact. Attempts to aggregate all associated elements of care to summarize the total costs of cognitive impairment to individuals, families, and the nation as a whole is complicated due to diverse data sources and differing measurement methodologies, among other factors. One of the most convincing estimates however can be generated for dementia care which results in the additional annual costs to the individual between $41,700 and $45,800 (adjusted for inflation in 2016 dollars) for informal care and care purchased in the marketplace (Hurd, Martorell, Delavande, Mullen, & Langa, 2013). Of that amount, the cost to individuals for their share of nursing home care totaled $15,300 and personal care in the home amounted to $6,800. Moreover, at over $119.6 billion for a national total of dementia-related direct health expenditures, the costs of dementia are similar to and in some cases exceed expenditures for heart disease and are substantially larger than the costs of cancer (Hurd et al., 2013). This is a conservative estimate given that dementia is present in a more restricted population than those people who experience significant cognitive deficits and come for treatment to the Emergency Department and Primary Care office. Because of this substantial economic impact, detecting and acting on cognitive compromise early is an economic imperative as well as a clinical necessity (Robinson, Tang, & Taylor, 2015) to improve the lives and care of people who live with dementia and their care providers (Barnett, Lewis, Blackwell, & Taylor, 2014). Furthermore, the presence of cognitive impairment has the potential to influence the effectiveness of doctor–patient communication, treatment adherence, the likelihood of medical follow-up, selection of appropriate medications, and likely medication side effects, thereby impacting overall health, which may add to caregiver burden and health costs.

Cognition should, therefore, be considered an important modifier of clinical outcomes and play an important role in clinical assessment (Weintraub et al., 2014). Screening for cognitive impairment may uncover underlying remediable conditions, or alert healthcare providers to increase surveillance for signs of progressive dementia. Identifying cognitive deficits and a dementing disease earlier allows for a more timely intervention, referrals to home and community-based services and social supports, and safeguarding health and financial management. The primary care setting is well positioned to provide early recognition of cognitive impairment and dementia; however, early symptoms of dementia, such as memory impairment, are not always apparent during a routine office visit and may not be voiced by the patient as a complaint. As a result, cognitive impairment and even frank dementia are often undiagnosed in primary care (Valcour, Masaki, Curb, & Blanchette, 2000; Boustani et al., 2005; Borson, Scanlan, Watanabe, Tu, & Lessig, 2006; Bradford et al. 2009). For example, a 2011 survey across 21 states found that 12.7% of adults over age 60 reported worsening memory problems in the prior 12 months, 35.2% of whom acknowledged accompanying functional difficulties, but only 32.6% of these individuals had discussed their concerns with a health care professional (CDC, 2013). Consequently, waiting for an expressed complaint may delay expeditious investigation of underlying causes of impairment and addressing individual needs. Cognitive screening and diagnosis in the ED and primary care settings could be beneficial to improve early detection and intervention and to initiate referrals to community-based care and assistance with treatment planning. In addition, identifying the presence of underlying cognitive impairment in older Emergency Department patients will have an impact on processes of care for them in the ED and impact clinical outcomes since dementia is the main risk factor for delirium and other atypical presentations of acute disease. Cognitive screening should not be limited to primary care and the ED as other specialty service clinics may also choose to implement cognitive screening protocols. For example, it is noteworthy that the American Diabetes Association recently recommended screening for early detection of mild cognitive impairment or dementia for adults 65 years of age or older at the initial clinic visit and annually as appropriate, recognizing the impact that cognitive impairment can have on diabetes management, comorbidities, and activities of daily living (ADA, 2018).

Although it has been widely recognized that the use of cognitive assessment tools increases the detection of cognitive impairment, which leads to early intervention, it is unclear how best to screen for cognitive impairment, including who should be screened, what tools should be used, who would carry out screening, and what sequence of procedures would follow screening (Cordell et al., 2013). Furthermore, it needs to be determined if a case-finding approach (i.e., using decision support tools to detect “at-risk” individuals) should be employed versus a mass population screening approach to detect early cognitive changes (Moyer & U.S. Preventive Services Task Force, 2014).

To examine how an interdisciplinary approach can best support the cognitive health of older Americans, the National Academy of Neuropsychology sponsored a Geriatric Summit on Assessing Cognitive Disorders Among the Aging Population on the 7th and 8th of December, 2017, in Denver, Colorado. The summit included an expert panel of speakers and participants representing the fields of neurology, neuropsychology, gerontology, geropsychiatry, primary care, emergency medicine, psychology, collaborative care, social work, and nursing, disease advocacy and policy groups, governmental agencies and providers, neuropsychological test publishers, and insurance providers. Participants came together to discuss the importance of cognitive screening and how best to implement screening in the assessment of older adults coming to the primary care and emergency room setting for medical care. This document summarizes the proceedings and content of the Summit and highlights the recommendations and key takeaways regarding unmet needs and future directions arrived at by the participants.

### Demographics of the Aging Population of the United States

The population of the United States is aging. While 9% of the population was over 65 years of age in 1960, that proportion is now 15% and will approach 25% by 2060 (Mather, Jacobson, & Pollard, 2015). The number of people over age 65 was approximately 49 million in 2016 and is projected to be 98 million by 2060 (Mather et al., 2015). During that same period, the number of people 85 years of age and older is expected to more than triple (Ortman, Velkoff, & Hogan, 2014; Mather et al., 2015).

The older population is also becoming more diverse with respect to race and ethnicity (Ortman et al. 2014). Nationally, all race and ethnic groups increased between 2015 and 2016 with the largest increase among the Asian and those self-identified as multi-race groups (U.S. Census Bureau, 2017). In 2014, the great majority (more than 75%) of those over 65 years of age were non-Hispanic white (Mather et al., 2015). The percent of people over age 65 who are Hispanic is expected to increase to 22% by 2060 (Mather et al., 2015). The increase in cultural, racial, and language diversity has important implications for the delivery of care to older adults since care is improved when language concordance exists between individuals and their healthcare care providers (Fernandez et al., 2011; Tang, Lanza, Rodriguez, & Chang, 2011; Jih, Vittinghoff, & Fernandez, 2015; Meuter, Gallois, Segalowitz, Ryder, & Hocking, 2015; Parker et al., 2017).

The regional and housing settings of older adults also have implications for health care delivery. The population over age 65 is not evenly distributed, tending to be located in certain parts of the country and to reach a higher proportion of the population in rural counties (Mather et al., 2015). Only about 4% of this population live in institutional settings such as nursing homes and a substantial proportion, as many as 56% of women and 30% of men over age 85, live alone in a personal home (Mather et al., 2015) and 79.5% of householders age 65 and older owned their own homes as of 2016 (U.S. Cenus Bureau, 2018). For people living in rural areas, and as the number of older people living alone increases, there may be an associated increase in the demand for services such as personal care and other home and community-based services (Mather et al., 2015) and for technologies that allow people to receive some care remotely.

### Importance of Cognition to Successful Aging

Maintaining cognitive health is important to successful aging. Age is the strongest risk factor for cognitive impairment (Blazer, Yaffe, & Liverman, 2015; Moyer & Force, 2014). Cognition is multifactorial and encompasses memory, attention, language, visuospatial skills, and executive functioning (Moyer & Force, 2014). Cross-sectional studies indicate that some aspects of cognitive ability (specifically, measures of fluid ability and processing speed) appear to steadily decline with age across all individuals, starting at around 30 years of age (Miquel et al., 2017). Usually, this decline does not affect everyday function, perhaps because people are able to maintain functional competence using cognitive abilities such as concrete knowledge that do not decline or even improve with increasing age (Salthouse, 2012). There are, however, numerous “normal” age-related changes in cognition that do affect the everyday function of older adults and place them at increased risk for functional and safety-related difficulties. While there is vast heterogeneity in the cognitive abilities of older adults, significant cognitive decline can contribute to errors in financial decision-making, impaired driving, and performance on technology-based tasks (Blazer et al. 2015). Avoiding risk factors for cognitive impairment and dementing diseases should be prominent goals for healthcare.

The ability to recognize cognitive impairment is important for many reasons. Some underlying causes of cognitive impairment, such as medication side effects or metabolic disorders, are potentially reversible. Early recognition of these conditions can lead to effective treatment and improved quality of life for both the person and their family. Cognitive impairment can affect adherence to treatment and lead to misuse of medications, which can lead to poor physical and emotional health outcomes (Stilley, Bender, Dunbar-Jacob, Sereika, & Ryan, 2010). Consequently, detection of cognitive impairment would allow healthcare professionals to modify their care pathways and provide education to improve treatment adherence (Arlt, Lindner Rösler, & von Renteln-Kruse, 2008).

Additionally, specific recognition of mild cognitive impairment syndrome (MCI) is important because it is a risk factor for progressive dementia, increasing its risk 10-fold (Belleville et al., 2017). Awareness of mild cognitive impairment can improve surveillance for and detection of Alzheimer’s disease or other dementing diseases, and allow people access to clinical trials and to better plan for the future, along with their families and caregivers (Sach-Ericsson & Blazer, 2015).

Sustained cognitive impairment and dementia are the main contributors to institutionalization in the older adult (Luppa et al., 2008) and represent substantial health care costs (The Healthy Brain Initiative: The Public Health Road Map for State and National Partnerships, 2013–2018, 2013). Given that healthcare resources are limited, informed decision making on health care management and efficient allocation of resources are important to minimize loss of opportunities (Handels, Wolfs, Aalten, Verhey, & Severens, 2013). For example, there is considerable research on redesigning systems of care intended to promote more efficient, but equally effective, allocation of treatment resources (Callahan et al., 2014; French et al., 2014; Long, Moriarty, Mittelman, & Foldes, 2014). These models commonly emphasize linkages with community resources and multi-agency coordination including health care providers, dementia care managers, internet-based care management protocols, and collaborative care planning with caregivers. Dementia is not the only area in which the force of economic reasoning has been introduced. The cost of disease, benefits of testing and intervention, as well as cost-effectiveness and cost–benefit of psychological assessment, were introduced and discussed at length by Yates and Taub (2003); Neumann (2004); Neumann and Greenberg (2009); and Neumann and Weinstein (2010).

### How to Detect Cognitive Impairment

The importance of identifying cognitive impairment is underscored by the inclusion, beginning in 2011, of assessment of cognitive function as part of the Medicare Annual Wellness Visit (AWV) benefit, as authorized by the Patient Protection and Affordable Care Act of 2010 (“Federal Register, Part II: Department of Health and Human Services, Center for Medicare & Medicaid Services,” 2010). Medicare is statutorily prohibited from paying for routine physical checkups with certain exceptions, the “Welcome to Medicare” exam, and the “Annual Wellness Visit” (AWV), which became available in 2011. The AWV includes and/or takes into account a health risk assessment and creates a personalized prevention plan that includes establishment of, or update to, the individual’s medical and family history, a list of the individual’s current providers and suppliers and medications prescribed; measurement of height, weight, body-mass index or waist circumference, and blood pressure; detection of any cognitive impairment; establishment or update of an appropriate screening schedule for the next 5–10 years; establishment or update of a list of risk factors and conditions (including any mental health conditions) for which interventions are recommended or underway; and furnishing of personalized health advice and referral, as appropriate, to health education or preventive counseling services or programs. The regulation implementing AWV states that in order to detect individuals at risk for cognitive impairment, the health professional should use direct observation in combination with information reported by the patient and/or concerns expressed by family and friends. In regard to cognitive screening, the U.S. Preventive Services Task Force (USPSTF) has noted that evidence to date is not sufficient enough to support screening of asymptomatic populations, nor is evidence sufficient to require the use of a specific tool. While no nationally recognized screening tool for detection of cognitive impairments exists at the present time, continuing efforts to identify a standardized screening methodology is needed. Still, relying simply on interview to detect cognitive impairment is insufficient given that cognitive deficits may go undetected by health care professionals using interview alone (Chodosh et al., 2004).

In 2013, the Alzheimer’s Association published recommendations for implementing the detection of cognitive impairment during the AWV through the use of objective cognitive measures rather than solely through voiced complaint or clinical impression (Cordell et al., 2013). The recommendations include an algorithm that begins with assessing every patient for signs or symptoms of cognitive impairment via medical records, observations, and concerns expressed by the patient or other informant. Administration of a cognition assessment tool would follow unless both the patient and a knowledgeable informant was present that affirmed cognition was normal (Cordell et al., 2013). If no knowledgeable informant is available at the visit, a brief validated cognitive assessment tool should always be used. Several brief (<5min) validated cognition assessment tools are recommended as options since no single tool has been identified as best for this purpose (Cordell et al., 2013). If resulting scores indicate concerns of cognitive impairment, the patient is referred for a full dementia evaluation (Cordell et al., 2013).

As mentioned above, in 2014, the USPSTF reviewed the evidence regarding screening the general population older than 65 years of age for cognitive impairment (Moyer & Force, 2014). The USPSTF arrived at an “I” rating (i.e., insufficient to assess the balance of benefits and harms of the service) regarding screening, concluding that there was insufficient evidence to weigh the benefits and harms of screening. Thus, routine screening of asymptomatic individuals may not be a common practice at present. The Veterans Administration has released a fact sheet for clinicians recommending against general screening and recommended the use of “dementia warning signs” consistent with the recommendations of the National Institute on Aging to identify patients who should be assessed for dementia (Clinician Fact Sheet: Detection of Dementia, 2011).

In 2015, The Gerontological Society of America (GSA) Workgroup on Cognitive Impairment Detection and Earlier Diagnosis published a report and recommendations to improve the assessment of older adults for cognitive impairment during visits with their primary care providers (The Gerontological Society of America Workgroup on Cognitive Impairment and Earlier Diagnosis: Report and Recommendations, 2015). The purpose of the GSA report was to achieve earlier diagnosis of Alzheimer’s disease and related dementias through increasing detection of cognitive impairment. The GSA recommendations include a process for approaching the AWV, summarized as KAER: Kickstart the conversation, Assess for cognitive impairment, Evaluate for dementia with full diagnostic workup if cognitive impairment is detected, and Refer to community resources and clinical trials or other research, if appropriate. The GSA recommended the routine use of one of the structured cognitive instruments recommended in the 2013 Alzheimer’s Association Report. Unlike the Alzheimer’s Association algorithm, it recommended a structured cognitive instrument in all situations, even if a knowledgeable informant confirmed a patient’s report that cognitive problems were not present.

## The Summit: Summary of Issues Addressed

Seven presentations introduced important aspects of multidisciplinary topics including how and by whom cognition is assessed, how assessment fits into current health care and reimbursement models, how electronic medical and health records can facilitate assessment, and how assessment might differ depending on setting such as emergency versus primary care. These presentations served as a basis for discussion after each talk and during the breakout groups that followed.

### Opportunities for Improving Cognitive Assessment in Older Adults

A substantial amount of research has been devoted to the diagnosis of and screening for Alzheimer’s disease, a form of progressive neurological disease causing dementia. In 1984, the National Institute of Neurological and Communicative Disorders and Stroke and the Alzheimer’s Disease and Related Disorders Association (NINCDS-ADRDA) Work Group published criteria to refine the clinical diagnosis of Alzheimer’s disease, noting that improved diagnosis was required for therapeutic trials; at least 20% of people diagnosed with Alzheimer’s were found not to have the disease upon autopsy (McKhann et al., 1984). The workgroup noted that no specific validated laboratory tests existed for Alzheimer’s disease. Over 30 years later, this remains true today, though NIA-AA is moving towards a biological definition of Alzheimer’s disease (Jack, Bennett, & Blennow, 2018) with the availability of CSF markers and pathologically validated brain imaging methods to detect with a high degree of certainty the presence or absence of amyloid plaque pathology in the brain with using FDA-approved radiopharmaceuticals (Clark, Schneider, & Bedell, 2011; Jack, Albert, & Knopman, 2011; Jack et al., 2013; Varma et al., 2018). Therefore, incorporating CSF and brain imaging biomarkers may be useful in the future.

Screening for cognitive impairment is quite different than screening for other medical conditions where a positive result may be diagnostic of a true condition (e.g., a colonoscopy for colon cancer). In the case of cognitive screening the Summit participants raised the concern that some may equate a positive screen with a diagnosis of dementia. Ashford and colleagues (2006) explain that in regards to cognitive impairment “screening tests determine when diagnostic tests should be considered” (p. 78). Detecting the presence of symptoms or signs of a disease does not require that formal diagnostic criteria be met. Consequently a positive screen should “only lead [s] to a recommendation of a second step in assessment” (p. 78) which may include imaging, neuropsychological testing, and activities of daily living assessments. Furthermore, there are many etiologies of cognitive impairment and dementia and differential diagnosis may require different evaluation methods. The distinction between tools for cognitive screening and diagnosis of dementia was a theme returned to frequently during the summit. Some important differences between screening (e.g., brief assessment tools in the clinical setting) and diagnostic tools (e.g., neuropsychological testing, PET scan, etc.) for evaluating cognitive decline and dementia are summarized in Table 1.

### Factors Influencing Diagnosis

It is well established that people with cognitive impairment and frank dementia often go undetected and are not properly evaluated to arrive at a diagnosis. The substantial rate of under-diagnosis may be due in part to the attitudes of some primary care physicians, who may be disinclined to diagnose something for which they feel there is a lack of useful interventions, as well as to discomfort on the part of healthcare providers and patients around the idea of dementia (Ofri, 2014). Additionally, most providers do not receive explicit training in how to identify cognitive impairment versus a diagnosis of dementia. However there are training programs available to help health care professionals identify some of the clinical differences between the major dementias (see: https://www.alz.org/health-care-professionals/dementia-diagnosis-diagnostic-tests.asp). Still, both the steps necessary to arrive at a dementia diagnosis and the disclosure of this diagnosis to a patient and their family can be time consuming and may require referrals for community resources. Patient and family/caregiver reluctance can also contribute to under-diagnosis; many people who are referred for further diagnostic tests do not follow up and many people would prefer not to know about a diagnosis for a condition with no available clinical treatment (Beck, 2012). Some feel an early diagnosis is “cruel” because, without a more effective treatment, the benefits do not outweigh the harms (Beck, 2012; Maguire et al., 1996), with potential harms to include a fear of loss of independence or of becoming a burden to family and friends. However, it should be noted that other services and supports, including home and community-based services, are available that may be beneficial to individuals with dementia and their caregivers.

In contrast, the World Alzheimer Report states that most people would wish to know about an Alzheimer’s diagnosis and that earlier diagnosis can improve the care and support of patients with Alzheimer’s disease (Prince et al., 2016). A survey of over 1,400 people found that most would take a test to predict a future disease, including Alzheimer’s disease, even if the test were not perfect and even in the absence of more effective treatments (Neumann et al., 2012). Many people with Alzheimer’s disease report they wish they had received an earlier diagnosis, before unrecognized dementia led to difficulties such as mismanagement of finances (Kolata, 2010).

Early recognition of cognitive impairment may have benefits in addition to identification of people who are at higher risk and should be monitored for Alzheimer’s disease. Cognitive impairment can increase the risk of cognitive side effects of medication and as a predisposing factor increases the risk of a delirium with acute illness, medication side effects, or procedures. Some medications may be contraindicated depending on the etiology of the dementia. In addition, cognitive impairment interferes with treatment adherence and increases patient frustration. Awareness of the presence cognitive impairment allows healthcare providers to better watch for these effects and to change care plans accordingly. Appropriate management of chronic diseases that require self-management, such as diabetes, also can be adversely affected if cognitive impairment is unrecognized or unaddressed (Sinclair, Girling, & Bayer, 2000). Modification of treatment and engaging family members and caregivers to assist in supervising medications and care can be critical to reducing medication errors and preventable hospitalizations.

### Who Should be Screened for Cognitive Impairment?

Although cognitive assessment is a benefit of the Medicare Annual Wellness Visit (AWV), the question of who should be screened outside of that forum and how they should be screened remains controversial. As stated earlier, the USPSTF reviewed the available science and concluded there was insufficient information to enable them to assess the relative benefits and harms of screening the general population of persons over 65 years of age for cognitive impairment (Moyer & Force, 2014). Therefore, the USPSTF made no recommendation regarding screening and noted that potential benefits of detection and early intervention have small effects or a lack of published evidence. Some potential adverse side effects associated with screening and current treatments were also noted (Moyer & Force, 2014). The Alzheimer’s Association has published recommendations for implementing the cognitive assessment benefit of the AWV, including the recommendation that standardized tools be used to make assessments because relying on clinical judgment is insufficient (Cordell et al., 2013). To help identify who should be screened during an AWV when there is no patient complaint and a knowledgeable informant is not present, a Dementia Screening Algorithm tool based upon dementia risk has been developed as an alternative to the Alzheimer’s Association recommended algorithm (Barnes et al., 2014), although this algorithm has not been prospectively evaluated.

The USPSTF report stated that, because evidence for the long-term benefits of available treatments on cognitive outcomes is not available, research into the effects of screening and early detection of mild to moderate dementia on decision making and planning could provide support for general population screening of individuals over age 65 (Moyer & Force, 2014). A single-blinded, randomized, controlled trial has been initiated to fill this knowledge gap identified by the USPSTF (Fowler et al., 2014). This trial randomly assigned individuals to be screened for cognitive impairment as compared to usual care, with those who screen positive referred for follow-up. Fowler and colleagues (2015) reported on an initial study of physician behaviors when physician groups were randomized to receive information about a patient’s cognitive function based on results of neuropsychological testing compared to physician groups with treatment as usual. Although the response was modest, physicians that received information about cognitive functioning were more likely to order blood tests to rule out reversible cognitive impairment and to document discussions about cognition with their patients. Patients from this group were also more likely to be on a cognitive-enhancing medication at follow-up. Rates of progression to dementia and cognitive outcomes were not different across groups; however, authors note that longer term benefits of this nature may not be identifiable with the relatively short study followup of 2 years compared with the long prodromal phase of Alzheimer’s disease. Results of this initial study can be extrapolated to show potential benefits of cognitive screening in a primary care setting.

The American Academy of Neurology has recognized the important role of the Medicare Annual Wellness Visit in detecting mild cognitive impairment. In its 2018 practice guideline “Update: Mild Cognitive Impairment” it recommends that when performing a Medicare Annual Wellness Visit, clinicians should not rely on historical report of subjective memory concerns alone when assessing for cognitive impairment and a brief, validated cognitive assessment instrument should be used (no specific instruments were recommended). The guidelines further recommend a formal clinical assessment and medical evaluation to identify and treat MCI risk factors that are potentially modifiable (Petersen, Lopez, & Armstrong, 2018).

Improvements in assessment technology could also assist in collecting the needed information. For example, researchers at Washington University in St. Louis, in collaboration with the Dominantly Inherited Alzheimer Network-Trials Unit, have created an Ambulatory Research in Cognition (ARC) smartphone app to increase precision and reliability of cognitive assessment to help in clinical trials. The app tests multiple aspects of cognition in short bursts multiple times per day; averaging the test results over time can avoid placing too much weight on a single assessment that may be influenced by variables such as time of day. The test was designed to minimize cultural and linguistic bias. Initial results indicate the app is feasible, reliable, and well tolerated (Hassenstab et al., 2017). However, this was in a younger population, so it remains to be seen how feasible this will be for older adults.

Summit participants discussed the importance of combating some physicians’ feelings of helplessness about assessing cognitive impairment and dementia and the need to provide information about benefits of early detection as well as solutions for patients who screen positive and their families and caregivers, as appropriate. They noted that the calculus of screening risks and benefits changes entirely if effective interventions for dementia are identified. Given the lack of solutions however, they emphasized the important need for the clinician to initiate conversations about brain health with the patient and their caregivers, and to provide referrals for additional education and supportive community services.

Participants also noted the importance of broadening the discussion to include all cognitive impairment rather than strictly focusing on Alzheimer’s disease. Though there may not be effective interventions for Alzheimer’s disease, many causes of cognitive impairment are reversible and patients can see substantial benefit if the source of cognitive impairment is identified early. An important aspect of early recognition of cognitive impairment is using tools validated with respect to language and cultural factors.

### Opportunities and Challenges of Assessing Cognition amidst Evolving Systems

The Centers for Medicare & Medicaid Services (CMS) is the largest purchaser of healthcare in the world, covering one-third of Americans through Medicare, Medicaid, and the Children’s Health Insurance Program. Like the private sector, health care service payments through CMS is moving from a fee-for-service model towards models that pay based on value rather than volume. These new models are person-centered and outcomes-based, with incentives for improved outcomes (such as reduced rates of hospital readmission) rather than volume and aim to support coordinated rather than fragmented care. In these new models, a single payment may be based on an episode of care rather than multiple payments being made for every visit and test that comprise an episode. Accountable Care Organizations (ACOs) and other integrated care models provide well-coordinated health care services with payment that is adjusted up or down based on domains including quality performance and reductions in the cost of care for the beneficiaries for whom ACO clinicians are accountable.

A goal of CMS is to empower patients and providers to make healthcare decisions. For coverage purposes, a diagnostic test is coverable if there is sufficient evidence that the results from that test is useful in medical decision-making, and that the management ideally leads to clinically meaningful, improved outcomes. Although no one test is universally accepted as the best test to detect cognitive impairment, several tests have been identified by the Gerontological Society of America (GSA) as satisfying criteria including being relatively fast to administer and having been recently validated in the United States (The Gerontological Society of America Workgroup on Cognitive Impairment and Earlier Diagnosis: Report and Recommendations, 2015). As described earlier, the GSA provides a toolkit with information, instruments, and materials including videos to support PCPs in implementing the KAER (Kickstart, Access, Evaluate and Refer) model. An important aspect of the KAER model is information to support communication about cognitive impairment and dementia, including the fact that conditions such as depression that often accompany dementia can be managed.

There are several payment vehicles for the assessment of cognitive impairment within the 2017 physician fee schedule. New codes describe integrated behavioral health models including the psychiatric collaborative care model that involves coordination between the PCP and a psychiatric consultant, behavioral health specialist, or behavioral health care manager. A new code, G0505, was introduced in 2017 specifically to pay for the assessment of patients with cognitive impairment, including dementia, and the creation of a care plan. Beginning in 2018 the G0505 code has been superseded with CPT code 99483 “Cognitive Assessment and Care Plan Services”. A requirement of this code is functional assessment and use of standardized instruments for staging of dementia that incorporates most of the appropriate features of a cognitive evaluation to arrive at a specific diagnosis. Importantly, this also sets the expectation that the caregiver is assessed for burden. Within the CMS Quality Payment Program (https://qpp.cms.gov), clinicians can be rewarded either through a merit-based incentive system, that includes quality measures specific to dementia care, or through participation in an advanced alternative payment model. These alternative payment models may serve beneficiaries with dementia and caregivers well over time.

In October 2017, the US Department of Health and Human Services sponsored a Research Summit on Dementia Care in which care models for people with a diagnosis of or at risk for dementia were presented. Common elements of many care models included enhanced care coordination, enhanced access to care, referrals and access to home-and community-based services, and caregiver services and support. A key takeaway is that a cognitive impairment care episode does not end with screening, but must include all follow-up services, and define next steps and who is involved in those steps.

The Improving Medicare Post-Acute Care Transformation (IMPACT) Act standardizes data across post-acute care settings such as long-term care hospitals and skilled nursing facilities to facilitate coordinated care and improve outcomes. The data that should be collected to best manage cognitive impairment have yet to be determined.

Summit participants discussed challenges associated with current means of compensation and payment codes. One issue that presents significant limitations is that Psychologists (including neuropsychologists, geropsychologists, rehabilitation psychologists, etc.) are not included in the definition of care providers for this service and so are unable to bill using the G0505/99483 code for assessment services on which they are the experts. Within the new outcomes-based billing models such as bundled services, such professionals may not be able to bill within a health system or practice group; thus, their expertise in assessing for cognitive impairment may be excluded due to lack of reimbursement mechanisms. Psychologists, particularly specialists within the psychology discipline, have critical skills and scientific knowledge that improve the cognitive screening procedure. They can have a key role in advising healthcare facilities about which screening measures are appropriate, how and where to set cutoff scores, teaching standardized administration of screening measures, and creating follow up trajectories for individual healthcare systems including EDs and primary care facilities. The proportion of alternative models employing team members who cannot bill Medicare directly is not known. In addition, it is not clear how to compensate for the increased cost associated with linguistic complexity when assessing and caring for a beneficiary with limited English proficiency.

### Predicting the Future with Clinical and Financial Data: Lessons Learned

Dr. Nirav Vakharia presented data from the Cleveland Clinic using an innovative approach to improve primary care, which may be relevant to screening for cognitive impairment. The Cleveland Clinic defines the population for whose care it is responsible to include the entire community, both those who seek medical care and those who do not. As an ACO, the Cleveland Clinic has been successful at reducing hospital readmissions and reducing costs through coordinating care for high-risk patients, including the use of intensive home care.

To find high-risk patients, Cleveland Clinic has focused on identifying and targeting conditions that lead to high, unplanned utilization (as opposed to conditions which are known to be associated with high utilization such as cancer). Clinical judgment was found to be inferior to a holistic model that included behavioral, functional, and social factors in addition to medical data. In this model, cognitive impairment, a behavioral factor, was the number one predictor of network leakage. Delirium was identified as a major factor that will likely predict risk of 30-day hospital readmission. In conclusion, financial, social, behavioral, and functional data can be integrated with clinical data to create risk models to identify patients who require increased coordination of care.

Summit participants discussed lessons that can be learned from the Cleveland Clinic experience regarding encouraging Primary Care Physicians and health systems to conduct a cognitive assessment even if it takes time from a limited appointment. To support this effort the Summit participants discussed the importance of demonstrating the benefit of screening by widely publicizing the data and its relevance; a strategy that has proven successful in the campaign to assess depression. Another consideration that was widely discussed is making screening easier or more efficient by allowing for medical assistants to conduct the screen and integrating the results directly into the electronic medical record. For example, in the Cleveland Clinic system, if a delirium screen is positive, the medical record will prompt the clinician to consider adding delirium to the problem list.

### Assessing Cognitive Impairment in the Emergency Department

The Geriatric ED Collaborative (Funded by the John A. Hartford Foundation and the West Health Foundation) is an interdisciplinary group including clinicians, social workers, and nurses whose goal is to implement guidelines for the care of older patients in emergency departments across the United States. In 2013, geriatric emergency department guidelines were approved and published by the American College of Emergency Physicians, the American Geriatrics Society (AGS), the Emergency Nurses Association, and the Society for Academic Emergency Medicine (Geriatric emergency department guidelines, 2014). These guidelines recommend routine cognitive screening of older ED patients for both chronic and acute cognitive impairment, (i.e. dementia and delirium) in those specialized emergency departments that have additional resources and established systems of referral.

Screening the ED population is quite different from screening the general population. In the general population, screening identifies mostly asymptomatic individuals who are at risk of a condition, while in the ED, the screening may more accurately be called case-finding with the purpose of identifying a condition among a pre-selected population of patients. Also, the time available for screening in the ED by a physician or other member of the interdisciplinary team is limited. Screening tools must be of the type that can be administered by other trained healthcare staff and must address the practical limitations of the environment such as assessing a patient who is lying on a gurney, is unable to sit up, unable to write, or even unable to vocally respond (Schnitker et al., 2015).

Implementation of cognitive screening in the ED faces many barriers. Routine universal cognition screening, in the ED may be challenging, even in individuals over the age of 65, due to environmental distractions, time and space constraints inadequate awareness and training of clinicians in recognizing cognitive impairment. Consequently, under ideal circumstances, routine cognition screening should be performed in a primary care or other professional outpatient setting. The suspicion of cognitive impairment or dementia should however be included in the patient’s medical record, so subsequent providers may be aware of the concerns of cognitive impairment and include that in their treatment planning. Identification of the patient’s primary physician, and effective communication between clinicians in the off-hours can, at times, be difficult. However, identification of cognitive impairment in the ED may improve subsequent care by improving patient adherence and follow-up thereby reducing reoccurring ED visits.

Finally, in the ED delirium must be assessed, as it represents a medical emergency. Similar to cognitive impairment in the primary care setting, delirium is common in the ED and yet may not be the focus of the emergency physician’s primary reason for attending to the patient and therefore not directly addressed (Elie et al., 2000; Hustey & Meldon, 2002; Lewis, Miller, Morley, Nork, & Lasater, 1995). This is further complicated by the inability of ED physicians to admit a patient with the diagnosis of delirium or “altered mental status” as these conditions are caused by other underlying medical emergencies. The recognition of delirium specifically and cognitive impairment in general in older ED patients is important given its prevalence (Hustey & Meldon, 2002; Wilber, 2006) and because it may be the principal symptom of a serious acute medical condition, impacting the clinical evaluation, patients’ understanding of medical information, and compliance with discharge instructions (Gerson, Counsell, Fontanarosa, & Smucker, 1994). Guidelines to implement geriatric-friendly processes of care in EDs recommend a two-step process for identifying delirium – a highly sensitive initial Delirium Triage Screen (DTS) followed by the use of a highly specific Brief Confusion Assessment (b-CAM) tool, conducted by a member of the medical team (e.g., nurse). A more in depth confirmatory assessment of potential delirium is then completed by another clinician (e.g, emergency physician, neurologist, neuropsychologist) for those screened positive at triage screen. However it is realized that not all EDs have the additional resources to support this level of assessment of the older patient (Geriatric emergency department guidelines, 2014; Han et al., 2013). Summit participants fully supported the need to recognize delirium as well as cognitive impairment and discussed the benefits of having an interdisciplinary team focused on geriatric care available. For example, systematic reviews indicate that adding a nurse practitioner specialist improves care (Ament et al., 2015; Burl, Bonner, Rao, & Khan, 1998) as well as a care manager who can convey critical information to the next provider and access a referral network of services if indicated. It was also acknowledged however, that consideration should be taken with the implications of ED clinicians introducing the diagnosing of dementia (chronic cognitive impairment) in older patients for whom they do not have an established doctor–patient relationship. Delivery of such diagnoses to patients and their caregivers, in a setting where the opportunity for interactive communication is often limited and would not allow for time to discuss the condition and its long-term management.

Consistent with recommendation above, the American Academy of Neurology has recommended that neurology be consulted in the ED and hospital for “high risk” cases in order to assess cognitive impairment using a delirium risk factor screening and preventive protocol (Josephson et al., 2017). The definition of “high risk” individuals for that measure includes both predisposing and precipitating factors defined as one or more of the following: age 65 years or older, known major/mild neurocognitive impairment, current hip fracture, severe illness (a clinical condition that is deteriorating or is at risk of deterioration), history of hypertension and/or alcoholism.

### Current Trends: A Managed Care Perspective

In the changing landscape of medical care reimbursement, there are several trends relevant to understanding how cognitive impairment assessment might be integrated in a managed care setting.

Many trends in managed care could be adapted to the care of cognitive impairment. Managed care is moving away from pay for volume to pay for value, and is evaluating providers and making these data available to providers and patients. Consequently, use of a cognitive screen to identify “at risk” individuals can be monitored and reported out as a component of a necessary health report.

Furthermore, much can be learned by viewing collaborative care service models, such as the CMS Psychiatric Collaborative Care Services Model (COCM https://www.cms.gov/Outreach-and-Education/Medicare-Learning-Network-MLN/MLNProducts/Downloads/BehavioralHealthIntegration.pdf). In this model, an enhanced rate is billed on a CPT code and the billing practitioner shares the fee with the rest of the team, which can include a behavioral health care manager, psychologist, or psychiatric consultant. Incorporating a multi-stage model in which positive results from either a performance screen or an informant-based assessment is followed up by specialists (e.g., neurologist or neuropsychologist) can be of great value (Galvin, 2018; Rosenbloom et al. 2016). In a recent study by Grober and colleagues (2016) a two-step cognitive screening approach using a brief battery of neuropsychological tests followed by a more extensive battery of neuropsychological tests when the initial battery indicated impairment was highly beneficial in identifying primary care patients with early dementia. Using a two-step approach coupled with a care manager in the clinical setting could potentially lead to even greater incremental cost-effectiveness.

Also in the pay-for-value models, providers may earn increased reimbursement or other benefits such as marketing support through improved clinician metrics (such as improved quality measured through wellness assessments, or reduced average cost per episode) or facility metrics (such as reduced readmission rate, or reduced average length of stay). Performance measures generally focus on three aims: improving health quality outcomes, improving overall population health, and reducing healthcare costs (Berwick, Nolan, & Whittington, 2008). A fourth aim, improving patient satisfaction, is highlighted in the Affordable Care Act, is sometimes included but is not always appropriate (Patient Protection and Affordable Care Act, 42 USC sec 1395ww 2015). For example, patient satisfaction may be difficult to measure for a provider whose work frequently involves communicating diagnoses with poor prognosis.

To assist providers, Optum Health developed an online tool (ALERT) to provide real-time feedback regarding clinical and claims data to identify outlier cases that may require increased interventions in order to avoid unnecessary medical interventions. The Summit participants promoted the notion that use of a standardized neuropsychological assessment instrument as part of a wellness assessment could potentially be adapted to assessment of cognition. To effectively use a tool such as ALERT for cognitive impairment, an appropriate metric will need to be identified, which can then be leveraged to demonstrate the utility of cognition assessment within the triple aim of improving population health, increasing patient satisfaction, and reducing per-capita health care spending.

Additionally, the Summit participants recognized that while it is important for health care systems to assess cognition in order to improve quality care and reduce expenses, it is also important to encourage patient and caregivers of the need for assessment through education. Consequently for cognitive impairment, this may include educating patients and caregivers about the benefit of early detection to avoid use of inappropriate medications and improve care of medical conditions and early diagnosis of the cause of cognitive impairment so a care plan can be established. Summit participants also discussed creative approaches to encourage people to seek screening, such as cognitive impairment literature that could be sent to consumers explaining the process and benefits of being screened. Primary care providers will also need to be educated regarding the value of assessment. In the experience of Optum Health, adding even a 30-second application to every PCP visit elicited pushback; thus, including screening for cognitive impairment would require education and payment incentives to encourage clinicians and medical systems to provide cognitive assessment.

### Using the Electronic Health Record in Assessment of Geriatric Patient Populations

The electronic health record (EHR) in general (also referred to as the CHR or Comprehensive Health Record) presents several potential roles in population health approaches, such as screening for cognitive impairment. Currently, the federal government requires that a minimum of 56 categories of data are shared between EHR platforms. This allows records to travel with the patient, improving the capacity to track changes in health parameters such as cognitive impairment over time. The EHR also allows the patient and all the clinicians involved in a patient’s care to access medical information in one place, increasing the opportunities for coordination of interdisciplinary care.

The EHR can empower patients to see their test results, find discharge and followup instructions, post questions to their care providers, and schedule reminders for medication. Dr. Alban from Epic indicated that patients access their EHR seven times more frequently than care providers, and that the largest group accessing EHRs are 50- to 60-year-olds.

The EHR can incorporate hundreds of variables, collecting social or other information in addition to medical information. In addition, many tools can be built into the CHR that would be relevant to screening for cognitive impairment, such as the ability to conduct remote video visits from within a linked application, or the addition of risk assessment tools that assess variables from the EHR and make recommendations about screening or further evaluation. Summit participants agreed that there needs to be standardized assessment tools and treatment plans that can be used across patient groups built into the EHR that allow for clear clinical pathways including when it is recommended to refer out to a cognitive specialist.

### Psychometric Challenges Associated with Assessing Cognition in Older Adults

One size does not fit all with respect to tests and tools used to assess cognition. The “best” assessment method will depend on what is being assessed and why, and any assessment method must be appropriately validated and results compared to an appropriate normative standard for the population being assessed. Results can vary substantially among different groups depending on age, education, intelligence, and social factors, making a single test with an absolute cutoff that could be applied to the entire adult population a challenging proposition. Psychometric considerations for selecting an assessment method include test reliability and validity, identification of correct normative reference values, test accuracy, and method of test delivery (such as computerized vs. face-to-face testing).

Assessing cognitive impairment on the surface may appear to be straightforward, but given that cognitive impairment ranges from mild cognitive diminishment to profound cognitive impairment, detection can widely vary. The most subtle of these conditions, MCI, may or may not be detected by others, particularly if the patient is exceptionally verbal. However, MCI does have adverse effects on cognitive function and should be reliably detectable by neuropsychological tests. The DSM-5 defines two types of impairment, mild neurocognitive disorder (which corresponds to MCI) and major neurocognitive disorder (which corresponds to dementia). These are defined as measured impairment in at least one cognitive domain, with performance in the 3rd–16th percentile placing a patient in the mild category and at or below the 3rd percentile in the major category. A recent analysis found a high false-positive rate in assessing for MCI, with almost 50% of the adults studied meeting criteria for mild neurocognitive disorder as defined in the DSM-5 (Holdnack et al., 2017). In another study, using the rigorous neuropsychological battery (NAB) assessment, over 1/3 of healthy adults would be classified as having mild neurocognitive disorder (Binder, Iverson, & Brooks, 2009; Iverson et al., 2008). Another study found 28% of people over age 75 met the criteria for MCI using only neuropsychological criteria that included low performance in at least two of ten tests, and in comparing two approaches to detect MCI, found discordant results in almost 37% of cases (Saxton et al., 2009). These studies highlight the importance of choice of assessment method and of test interpretation. Interpretation algorithms should be optimized to meet the objectives of the assessment, and may need to be adjusted based on whether the false-positive (a test result that incorrectly indicates cognitive impairment) or false-negative (a test result that incorrectly indicates the absence of cognitive impairment) rate should be minimized so that selection criteria serves as an improvement over subjective complaints and clinical judgment (see Edmonds et al., 2014, 2015, 2016). It is important to keep in mind that identifying precise criteria is difficult because the performance of healthy and clinical groups will overlap.

To obtain valid data, tests must be delivered in a person’s primary language, and must be normalized within a representative population and results compared to an appropriate baseline. The participants recognized the overlooked fact that according to the Census Bureau from the 2013 American Community Survey (ACS), 61.8 million Americans do not speak English at home (https://www.census.gov/acs/www/data/data-tables-and-tools/). Because cutoffs for identifying impaired performance will be defined relative to normative reference values it is essential to have an accurate measure of the distribution for a given population. Normative results can vary depending on reading ability, occupation, language and culture. Summit participants also emphasized the fact that many additional factors affect the results of a cognitive assessment including pain, lack of sleep, fatigue, stress, anxiety, and depression, all of which must be considered when interpreting results.

Another consideration that must be acknowledged is that not all cognitive impairment is permanent and impaired cognitive performance frequently returns to normal over time (Brooks, Iverson, Holdnack, & Feldman, 2008). Thus, future algorithms need to become more sophisticated and adjust criteria for impairment dependent upon specific patient characteristics (Brooks, Iverson, Feldman, & Holdnack, 2009).

While the summit participants were fully supportive of the need for cognitive screening, the consensus was that a cognitive screening program must consider time and cost, the training and expertise required of those administering the screening, the sensitivity and specificity of the screening method, and how or whether to monitor change in cognition over time. The group recognized the potential for computerized testing which presents several advantages such as ease of use, standardization, and the potential for automated scoring and interpretation, though results can depend on computer literacy (Iverson, Brooks, Ashton, Johnson, & Gualtieri, 2009). The group however also recognized the need to consider the assessment from the perspective of the patient who may be computer-adverse or find the hospital room ED setting too distracting. In those cases, paper and pencil testing or at-home evaluations may be preferable.

## Breakout Groups

Following the presentations and discussions, the summit attendees broke into four focused groups to discuss the following topics:

- The implementation of screening protocols in primary care and emergency department settings
- Identification of “at risk” patients: the role of registries and what occurs when a person tests positive on a screen
- Compensation for services – moving from fee-for-service to full risk and value-based payment models
- Next steps: creating clinical pathways for persons identified as potentially having a cognitive disorder

Further key takeaways from each group are described below.

### The Implementation of Screening Protocols in Primary Care and Emergency Department Settings

The Summit group determined that an effective cognitive screening protocol will need to differ based on clinical setting. The group acknowledged that cognitive impairment in the ED typically falls into two important and ED-specific categories: chronic, i.e. a likely dementing disorder, which impacts the care that patient receives by influencing ability to gather an accurate history and perform an informative physical exam and the person’s ability to understand and participate in processes of care and discharge planning; and acute, i.e. delirium, which is an essential ED presentation since it usually represents a symptom of an acute occasionally life-threatening medical condition. Strategies for identifying them are significantly different in the ED, however use of an initial and quick screening measure (preferably completed within one minute) could include an initial triage determination followed by an alternative screen in cases where delirium is suspected. Stress, pain, and other factors frequently present in ED patients and can affect performance on cognitive screens; thus, alternative factors must be considered when assessing cognitive impairment in the ED. In the primary care setting, a test may be able to take as long as 5min and should be considered as part of the AWV or any visit when there is a suspicion of memory concern. In the primary care setting, novel approaches such as treating cognitive screening as a laboratory test (performed outside of the PCP office) may present an alternative to finding time to conduct screening during an office visit.

In both settings, screening may be made more efficient by incorporating information about demographic and medical risk factors from the patient’s EHR. A two-tiered approach could be considered in which initial demographic and medical risk factors apparent in the EHR are factored in to the algorithm to identify those with at high risk for cognitive impairment (Walters et al., 2016). Once high-risk candidates are identified, they can be referred to a cognitive specialist (neurologist geriatrician, geriatric psychiatrist, or neuropsychologist). Collateral information from informants also should be considered when available.

Risk factors that might identify high-risk patients to guide cognitive screening in the primary care and ED settings include age, history of stroke, educational attainment less than 12 years, depressive symptoms, presence of diabetes mellitus, substance abuse, and caregiver assistance needed for finances and/or medication (The Gerontological Society of America Workgroup on Cognitive Impairment and Earlier Diagnosis: Report and Recommendations, 2015). Other neurological diseases, such as a history of traumatic brain injury, multiple sclerosis, epilepsy, and Parkinson’s disease are at high risk of cognitive impairment and could be included. Additional less-obvious factors that were identified included medication non-adherence, frequent calls to the PCP office, and after visit summary confusion. The summit participants also recommended future research to identify the optimal risk factors that could identify high-risk individuals and can be obtained from data in the EHR.

Also, in both the ED and primary care setting, a care pathway should be readily available that identifies clear next steps after positive findings. It may be inappropriate to rely on a single positive cognitive screen for dementia especially if performed in a less-than-ideal setting, the ED. A strategy similar to that followed for identifying hypertension might be followed. A diagnosis of hypertension requires a high blood pressure reading on at least two visits. A separate visit to verify cognitive impairment with either another test instrument (for example the more extensive 30-point, 10–15min Montreal Cognitive Assessment) or an alternative version of the same screening instrument would also allow a clinical discussion and interpretation before initiating a full cognitive evaluation. Summit participants recognized that to encourage screen usage it would be helpful to have more evidence that screening improves patient outcomes (such as reducing return visits to the ED or improving treatment adherence and care follow-up). In both settings, the care team should understand the benefit behind why the screen is being done.

In all cases, the patient should be screened in their preferred language. If this is not possible, a risk factor-based algorithm may be preferred to administering a screening test without language concordance because language discordance will likely lead to false positive results. Language concordance has been demonstrated to improve outcomes, decrease medical errors, improve treatment compliance, and decrease costs (Fernandez et al., 2011; Jih et al., 2015; Meuter et al., 2015; National Standards for Culturally and Linguistically Appropriate Services in Health Care, 2001; Parker et al., 2017; Tang et al., 2011). The summit group recommended that hospital systems identify the top five languages used by their population and prepare for screening within these languages. This will involve identifying linguistic assets and implementing appropriate staffing, instrumentation, and care pathways.

Additionally, the group recommended that screening tests be shown to be reliable and validated in different target populations, considering cultural factors in addition to language factors (Ardila, 2005; De Jesus-Zayas, Buigas, & Denney, 2012; Romero et al., 2009).

A screening test for cognitive impairment should be able to be administered by any trained member of healthcare team. Some levels of screening, such as The Eight-Item Informant Interview to Differentiate Aging and Dementia (AD8 dementia screening interview), or collecting written information from the patient, could be performed by medical technicians and office staff. With proper training, medical technicians could complete other screening assessments.

The group recommended that the screening be characterized as a measure of brain health since screening tests are not diagnostic. It was also emphasized that having a geriatric trained behavioral health worker, or case manager (or a repurposed member of the existing team) to walk the patient through the screening and subsequent follow-up could reduce recidivism and decrease utilization. This will be increasingly important as compensation moves to full risk reimbursement models. Identifying an advanced, technologically effective but least-cost method of screening and follow-up should be a priority area of behavioral workforce policy (Garety et al., 2018; Nemec & Chan, 2017; Raney, Bergman, Torous, & Hasselberg, 2017).

The group stressed that the results of cognitive screening results should always be integrated into the EHR in a manner that allows data to be captured for quality metrics, population health management, syndrome surveillance, and research. The EHR may also be capable of scoring screening tests and offering suggested interpretation, optimizing clinician time. Screening result interpretation should be available to all of a patient’s providers because as a dominant comorbidity cognitive impairment influences the management and choice of drug treatment for all other conditions. This can be accomplished by utilizing EHR problem lists to identify cognitive impairment, health maintenance tools and alerts, and condition registries. The group recommended that EHRs create modules that include confusion and cognitive impairment screens so that hospital systems do not have to individually create such tools.

### Identification of “At Risk” Patients: The Role of Registries and What Occurs When a Person Tests Positive on a Screen

The objective of screening for cognitive impairment is to improve patient care and outcomes for people with cognitive difficulties. The group emphasized that screening is not diagnostic; it provides important information to the clinician to make informed decisions and to consider next step pathways. Ultimately the goal of screening is to lead to improved patient care. In turn, screening combined with additional sources of information can lead to identifying remediable causes of cognitive impairment that can be medically addressed; transition planning that can better ensure patient safety and adherence to recommended treatment or follow up care; reduction in hospital, PCP, and ED use; and finally provide education, planning, and support to the patient and family/caregiver to improve quality of life.

Identifying “at risk” patients differs depending on the medical setting. In the ED the primary goal may be to identify risk to prevent, recognize, and treat delirium. In this setting, the patient may be best assessed by a primary ED nurse and if delirium is identified, a pathway including evaluating underlying causes and deploying a care plan can be pursued. If there is a history of cognitive impairment or cognitive complaints, a delirium prevention protocol can be implemented (Josephson et al. 2017; Marcantonio, 2017). In the primary care setting, the primary goal may be to optimize adherence to personalized care plans. Assessment may occur during the AWV or next care visit based on risk factors or response to brief targeted questions. Positive screening results would lead to a pathway involving further assessment and additional support. While some patients may not want to be told that they require a workup for cognitive impairment and a referral to a cognitive specialist such as neurologist, studies have shown that patients experiencing cognitive problems favor being told that they have a cognitive impairment and the possible cause of their problems (Elson, 2006).

A knowledge gap identified by the Summit group is what information to leverage from the EHR/CHR to identify at risk patients in the primary care and ED settings. Registries comprised of databases may be used to fill this gap. The group however provided some cautionary thoughts about this approach and the ethics of identifying and classifying patients as “at risk” without their knowledge.

The group agreed that the benefits of data-driven algorithms to identify people at risk for cognitive impairment outweighed the potential unintended consequences. The EHR can assist in this process by ensuring that all older adult patients, whether a member of an ACO or not, are evaluated on an annual basis. The resulting cognitive screening data can then be added to other medical and non-medical, non-cognitive information such as, the presence of diabetes, medications that impact cognition, age, reason for visit, and the number of phone calls or messages to clinicians, as well as psychological, functional, and social areas. Once collected the data can then be used to build an algorithm to stratify patients on the basis of risk for cognitive decline. Additional patients would be screened based on complaint of a change in cognition reported by the patient or family member. Use of the EHR in this fashion can also minimize the implicit bias that can be present in the primary care setting which affects the rates at which different patients are referred to specialist for follow up.

The summit group agreed that once an algorithm is empirically determined it should be reexamined to ensure that it is providing a valid procedure at each location or whether geographic distinctions need to be accounted for. The group asserted that ideally there needs to be a care pathway to direct follow up after screening, including clear guidelines on how often follow up assessments need to be conducted based upon a screening result in combination with other factors. Furthermore, health systems should aspire to include some type of care team follow-up or contact via phone call.

### Compensation for Services – Moving from Fee-for-Service to Full Risk and Value Based Payment Models

This group emphasized that screening is different from assessment or diagnosis. Screening as part of a collaborative care pathway fits well into multispecialty, longitudinal episodes of care models and bundled payment models. However, the care team should include non-physician clinicians not eligible for Medicare reimbursement for this service (e.g., psychologists) because they have expertise in cognitive assessments. These care and payment models that bundle services over longitudinal episodes or that are delivered by a team of professionals may deliver improved health outcomes for dementia patients and their caregivers, but require further study.

Presently insurance companies are contending with bottlenecks to care, as demand increases while simultaneously access to care decreases. Incentives for the system to evaluate more patients could incorporate care pathways and opportunities for physician “extenders” to provide care to reduce physician burden and facilitate care across settings. For cognitive screening, this could involve offering higher reimbursement for initial evaluations for at-risk individuals, and additional payments for practices furnishing higher levels of care. Telehealth services might also be leveraged to reduce caregiver burdens, especially when delivering care to patients living in rural or isolated settings, or when language concordance is of issue. Early detection or diagnosis has the potential for cost saving and redirection of resources to those with the most pressing health related problems. This possibility deserves further study. Alberdi, Aztiria, and Basarab (2016) have recently addressed this as a cost saving issue with respect to the early diagnosis of Alzheimer’s disease. As for dementia, in a letter to the editor discussing the benefits of early diagnosis, the editor of the *International Journal of Geriatric Psychiatry* asserts that “ … interventions can take place to avoid crises” and “[the] economic benefit–avoiding unnecessary admissions and institutionalization–is well known” (Burns, 2012, e3556). If “planning” has the positive attributes suggested for most future activities it would appear that early detection of dementia would clearly help avoid excessive and unnecessary costs.

### Next Steps: Creating Clinical Pathways for Persons Identified as Potentially Having a Cognitive Disorder

The final group noted that designing clinical pathways assumes the correct population was screened using a valid and accurate test. As stated above, reasons for potential false positives should always be included in the interpretation of results and considered before proceeding.

Immediate next steps will depend on the clinical setting and the severity of the screening result. In the ED, an established clinical care pathway should be implemented for delirium prevention and delirium treatment that includes a strategy for assessing potential causes as well as for the management of the condition. In this regard the pathway could include determining acute care needs and the need for admission but also remind providers to establish surrogate decision makers, and to consult with a cognitive specialist on the team. When a screen for cognitive impairment is positive but delirium is not present, the care pathway would include obtaining information from available sources such as collateral informants. A care manager or social worker might be activated to direct the patient to appropriate resources and help ensure compliance with follow-up plans.

In the ED, positive screen results affect clinical decision making in the design or modification of care plans taking into account the patient’s capacity for decision making or understanding, assessment of safety for discharge, identification of discharge considerations such as who will be helping to care for a patient, providing written information to the patient and family/caregiver and referrals to appropriate resources, and increasing follow up. A positive screen may also trigger assessment by an interdisciplinary team, referral to follow-up for cognitive disorder or dementia, or referral to outpatient treatment if behavioral issues are predominant. Assessment via an interdisciplinary team, including social work and clinical care managers, may also generate referrals to home and community-based services and social supports.

In the primary care setting, a positive screening result would result in implantation of a care plan in line with existing guidelines from sources such as the AGS and Alzheimer’s Association or specific to the healthcare system. The plan should include confirming cognitive impairment on a second visit, identifying and treating remediable causes of cognitive impairment, and determining a specific protocol for how cognition would continue to be monitored over time, and when a referral to a specialist is indicated. Specialist referrals may be triggered by atypical presentations and behavioral dysregulation and would rely on recommendations from existing guidelines. Specialist referrals also would be appropriate if performance and interpretation of a cognitive evaluation to determine the cause of impairment is outside the provider’s scope of practice. Specialists such as neurologists, geriatric psychiatrists, geriatricians, and neuropsychologists can be called on to clarify diagnosis, conduct further testing to inform treatment and track response to treatment, and to address emotional distress and environmental adjustment, provide education, and caregiver support. Specialist availability will depend on available resources, so any care plan must provide flexibility for settings with no access to certain specialists. This group emphasized the value for patients with cognitive impairment of care managers who can organize care, provide educational information and materials, direct patients to community resources and other social supports, and address functional needs that can minimize risks of decline.

Many types of educational materials can be provided to patients and their families and caregivers. The information should be adapted as needed for patient and caregiver. Helpful materials may include:

- Condition-specific information (e.g., symptoms, expected progression, etc.)
- Caregiver resources and support (includes respite and social support)
- Community Resources (e.g., adult day health, community/senior centers, adaptive sports programs, paratransit services, Neuro Recovery centers, etc.)
- How to manage functional limitations, supervision needs, and behavioral changes
- Safety Issues within home and community
- Advance Care Planning (e.g., POA, Living will, Guardianship)
- Referral to existing information sources (e.g., Alzheimer’s Assoc., Council on Aging, AARP, etc.)
- Empirically validated options to promote positive cognitive health (e.g., exercise, social and community involvement, healthy diet, cognitively stimulating activities, etc.)
- Information about sleep – importance of healthy sleep patterns and risks for cognition related to disrupted sleep

## Key Takeaways and Knowledge Gaps

In conclusion, the Summit attendees emphasized the power of an interdisciplinary approach to improve care and the significant range of expertise available when professional organizations come together. To summarize, the key takeaway conclusions are listed in Table 2.

Furthermore, the Summit participants identified knowledge gaps that exist which can be the basis for future collaborative research endeavors (see Table 3).

For economists, an increase in the number of options or an expansion of the data set often provides a basis for a more efficient and quite possibly a lower cost solution to accomplishing an objective. Even where a final resolve is not available, early detection at least could improve the prospects for better quality of life associated with a cognitive impairment diagnosis. With respect to Alzheimer’s disease, Barnett and colleagues (2014) studied early identification of cognitive loss within a cost-effectiveness context and found that net economic benefits were reduced by approximately 17% for every year of delayed identification.

Finally, the Summit participants suggested follow up meetings to track success in the assessment of cognitive disorders among the aging population. The participants expressed hope that the proceedings from this meeting will provide practitioners and health systems with useful guidelines to consider when developing clinical practice pathways. Given the complexity of clinical decision-making, diversity of services required, and the need for coordination of services for the person experiencing cognitive impairment, implementation of a team-based, collaborative care approach may best serve to improve the identification and care of people with cognitive disorders.

## Figures and Tables

**Table 1.** Differences between screening and diagnostic tools.

	Cognitive screening measures	Diagnostic tests for dementia

Purpose of test	Detect potential disease indicators	Establish presence or absence of a specific disease
Target population	Large number of individuals selected on the basis of demographic or clinical characteristics who are not previously diagnosed with the condition of interest	Symptomatic individuals, or those at high risk
Test characteristics	• Simple, acceptable to patients and staff • Inexpensive; the benefit must justify the cost of screening large numbers of individuals	• May be invasive; precision of test weighted more than its patient acceptability • May be expensive; cost is justified as necessary to establish diagnosis
Positive result threshold	Set to achieve high sensitivity (maximize potential positives)	Set to achieve high specificity (minimize false negatives)
Implication of positive result	Suspicion of disease; in combination with other risk factors provides reason for additional follow up	Provides definite diagnosis and thus prognosis and identification of appropriate management

**Table 2.** Key takeaway conclusions.

• There is a need to educate the public regarding the difference between *screening*, which identifies individuals at risk within an asymptomatic population, and *diagnosis* of a specific condition such as delirium or a dementing disease.
• It is important to emphasize that cognitive impairment screening is a measure of brain health, which needs to be monitored regularly in at-risk individuals to determine the fidelity of brain functioning.
• Cognitive impairment in older adults has multiple possible causes, including medical and psychiatric conditions, such as endocrine and metabolic conditions, chronic pain, depression, sleep disturbance, medication side-effects, delirium, and brain diseases causing dementia, with Alzheimer’s disease and MCI being the most common.
• Cognitive impairment is a clinically dominant comorbidity. Cognitive impairment is so serious that it overshadows the management of other health problems. It influences the effectiveness of doctor–patient communication, treatment adherence, the likelihood of medical follow-up, the selection of appropriate medications, and likely medication side effects.
• Cognitive evaluation to determine the causes and remediable factors contributing to impairment is necessary to guide appropriate choice of medications and management.
• Collaborative care models that include the expertise of specialists in the area of cognitive assessment (i.e., neuropsychologists, neurologists and geropsychiatrist) may be cost-effective and provide better quality care. In the emerging value over volume payment models, inclusion of cognitive specialists fits well into new team-based payment models that emphasize overall wellness.
• The EHR presents a great deal of promise for risk stratification modeling and for monitoring changes in cognitive screen performance over time. EHR automated tools for assessing and recording the results of individual’s cognition over time need to be developed.
• There is a need to increase awareness of identifying risk factors beyond medical data that include social, behavioral, and functional information.
• No one size fits all when assessing for cognitive impairment. It is important to recognize that the goals and means of cognitive assessment depend on the clinical setting and differ between the ED and the primary care environment.
• There is an important role that care managers or coordinators play in ensuring that people stay on a care pathway, and may also increase patient and caregiver satisfaction.
• There are deficiencies in health services in rural and economically disadvantaged America, resulting in a large gap in access to care and differences in resources such as care coordinators and cognitive specialists.
• Assessment of cognition must be done in a linguistically and culturally appropriate way to obtain meaningful results.
• There is a great need to increase advocacy regarding Medicare coverage and payment for a range of services and supports for beneficiaries with cognitive impairment (for example, including reimbursement for psychologists on interdisciplinary teams).

**Table 3.** Identified knowledge gaps.

• The need for research to determine whether “best practice” algorithms will guide risk stratification models and improve detection of cognitive impairment.
• Empirical determination of the best screening tools to use to assess cognitive impairment when taking in to consideration patient and provider acceptability, cost, time, sensitivity and specificity.
• How best to factor in language and cultural determinants when screening for cognitive impairment.
• How best to factor in a person’s age, intelligence, and education when interpreting screening and assessment results.
• What care models are most effective, time and cost efficient when conducting cognitive screening in the absence of clinical signs of cognitive disorder.
• How to consider repeated screening and the use of baseline data against which future assessments can be compared.
• What ways the EHR can best be leveraged to identify at risk individuals and document for all providers the cognitive health status of their patients.
• How best to utilize “smart technologies” and how to integrate these technologies with traditional medical data.

## Summit Participants

Deb Adler^1^, Christopher Alban, MD, MBA^2^, Mark Bondi, PhD^3^, Michelle Braun, PhD^4^, Xavier Cagigas, PhD^5^, Morgan Daven^6^, Robert L. Denney, PsyD^7,8^, Lisa Drozdick, PhD^9^, Norman L. Foster, MD^10,11^, Ula Hwang, MD^12–15^, Laurie Ivey, PsyD^16^, Grant Iverson, PhD^7,17^, Joel Kramer, PsyD^18^, Laura Lacritz, PhD^7,19^, Melinda Lantz, MD^20^, Lisa Latts, MD, MSPH, MBA^21^, Shari M. Ling, MD^22^, Ana Maria Lopez, MD^23–26^, Michael Malone, MD^27,28^, Lori Martin-Plank, PhD, MSN, MSPH, RN^29^, Katie Maslow, MSW^30^, Don Melady, MSc(Ed), MD^31–33^, Melissa Messer^34^, John Meyers, PsyD^7^, Charles E. McConnel, PhD^19^, Randi Most, PhD^36^, Margaret P. Norris, PhD^37^, William Perry, PhD^7,85,39^, Neil Pliskin, PhD^40^, David Shafer, MBA^41^, Nina Silverberg, PhD^42^, Tresa Roebuck-Spencer, PhD^43,44^, Colin M. Thomas, MD, MPH^45^, Laura Thornhill, JD^46^, Jean Tsai, MD, PhD^10,47^, Nirav Vakharia, MD^48^, Martin Waters, MSW^49^

Organizations Represented

Alzheimer’s Association, Chicago, IL

AMA/CPT Health Care Professionals Advisory Committee, Chicago, IL American Academy of Clinical Neuropsychology (AACN), Ann Arbor, MI American Academy of Neurology (AAN), Minneapolis, MN

American Association of Geriatric Psychiatry (AAGP), McLean, VA American Association of Nurse Practitioners (AANP), Austin, TX American Board of Professional Neuropsychology (ABN), Sarasota, FL American College of Emergency Physicians (ACEP), Philadelphia, PA American College of Physicians (ACP), Philadelphia, PA

American Geriatrics Society (AGS), New York, NY

American Psychological Association (APA), Washington, DC Beacon Health Options, Boston, MA

Canadian Association of Emergency Physicians, Ottawa, ON, Canada Collaborative Family Healthcare Association (CFHA), Rochester, New York Gerontological Society of America, Washington, DC

Hispanic Neuropsychological Society (HNS), Los Angeles, CA

W. Perry et al./Archives of Clinical Neuropsychology (2018); 1–21

IBM Watson Health, Denver, CO

International Federation of Emergency Medicine, West Melbourne, Australia

International Neuropsychological Society (INS), Salt Lake City, UT National Academy of Neuropsychology (NAN), Denver, CO

Optum of UnitedHealth Group, Minneapolis, MN Pearson, New York City, New York

Psychological Assessment Resources, Inc, Lutz, FL Society for Clinical Neuropsychology, Washington, DC U.S. Department of Veterans Affairs, Washington, DC

*Please note that participation in the Summit does not constitute organizational endorsement of this report

Department of Neurology, University of Utah; ^12^Geriatric EM Section, American College of Emergency Physicians (ACEP); ^13^Department of Emergency Medicine; ^14^Icahn School of Medicine at Mount Sinai, Geriatric Research Education, and Clinical Center, James J. Peters VAMC Geriatric EM Section; ^15^American College of Emergency Physicians (ACEP); ^16^Collaborative Family Healthcare Association (CFHA); ^17^Neuropsychology Outcome Assessment Laboratory and Director, Massachusetts General Hospital for Children Sports Concussion Program, Harvard Medical School; ^18^International Neuropsychological Society (INS); ^19^UT Southwestern Medical Center; ^20^American Association of Geriatric Psychiatry (AAGP); ^21^IBM Watson Health; ^22^Centers for Medicare and Medicaid Services (CMS); ^23^American College of Physicians (ACP); ^24^Health Equity and Inclusion, University of Utah Health Sciences Center; ^25^Cancer Health Equity, Huntsman Cancer Institute; ^26^University of Utah School of Medicine; ^27^American Geriatrics Society; ^28^Aurora Senior Services, Aurora Health Care; ^29^American Association of Nurse Practitioners (AANP); ^30^The Gerontological Society of America (GSA); ^31^Schwartz/Reisman Emergency Medicine Institute, Mount Sinai Hospital, University of Toronto; ^32^Canadian Association of Emergency Physicians; ^33^International Federation of Emergency Medicine; ^34^Research & Development, Psychological Assessment Resources, Inc. (PAR); ^35^NAN; ^36^American Board of Professional Neuropsychology (ABN); ^37^American Psychological Association (APA); ^38^NAN; ^39^University of California, San Diego; ^40^University of Illinois at Chicago; ^41^Pearson; ^42^Alzheimer’s Disease Centers (ADC) Program, National Institute on Aging (NIA); ^43^NAN; ^44^Jefferson Neurobehavioral Group; ^45^Department of Veterans Affairs; ^46^Regulatory Affairs, Alzheimer’s Association; ^47^University of Colorado, Denver Health; ^48^Cleveland Clinic; ^49^Clinical Innovation and Thought Leadership, Beacon Health Options; ^50^Golden Bioscience Communications
